# Pre-diagnostic ALSFRS-R slope provides limited incremental information about the variability in subsequent functional decline in amyotrophic lateral sclerosis: a population-based study

**DOI:** 10.1007/s00415-026-14177-2

**Published:** 2026-10-08

**Authors:** Rosario Vasta, Andrea Calvo, Cristina Moglia, Antonio Canosa, Umberto Manera, Giulia Gianferrari, Elisabetta Zucchi, Ilaria Martinelli, Gabriele Mora, Sara Cabras, Francesca Palumbo, Enrico Matteoni, Alessandra Maccabeo, Giorgio Pellegrino, Emilio Minerva, Daniela Pascariu, Stefano Callegaro, Fabrizio D’Ovidio, Letizia Mazzini, Fabiola De Marchi, Jessica Mandrioli, Adriano Chiò

**Affiliations:** 1 ALS Center, Department of Neuroscience “Rita Levi Montalcini”, University of Turin, via Cherasco 15, 10126 Turin, Italy; 2 Neurology 1, AOU Città della Salute e della Scienza di Torino, Turin, Italy; 3 Institute of Cognitive Science and Technologies, National Research Council, Rome, Italy; 4 Department of Biomedical, Metabolic and Neural Sciences, University of Modena and Reggio Emilia, Modena, Italy; 5 Department of Neurosciences, Azienda Ospedaliero-Universitaria Di Modena, Modena, Italy; 6 Department of Translational Medicine, University of Eastern Piedmont, Novara, Italy; 7 Azienda Ospedaliero Universitaria Maggiore della Carità, Novara, Italy

**Keywords:** Amyotrophic lateral sclerosis, ALSFRS-R, Disease progression, Clinical trials

## Abstract

**Background:**

The pre-diagnostic ALSFRS-R slope is frequently used in a clinical trial design and clinical practice under the implicit assumption that it reflects subsequent functional decline. We assessed the association between the pre-diagnostic ALSFRS-R slope and subsequent functional decline.

**Methods:**

The Piemonte and Valle d’Aosta ALS Registry (PARALS) was used as the primary cohort. The associations between pre- and post-diagnostic slopes at 6, 12, and 18 months were examined using correlation coefficients and multivariable linear regression. Linear and quadratic mixed-effects models used all ALSFRS-R assessments obtained during the first 18 months to evaluate longitudinal trajectories and quantify the incremental contribution of the pre-diagnostic slope. All analyses were replicated in the Emilia Romagna Registry for ALS (ERRALS).

**Results:**

A total of 2,222 patients from PARALS and 1,543 from ERRALS were included. In PARALS, the pre-diagnostic ALSFRS-R slope showed only a modest monotonic association with post-diagnostic functional decline (Spearman’s *ρ* = 0.19–0.30) and explained 6.1% of its variance at 6 months, with this proportion decreasing thereafter. Quadratic mixed-effects models estimated that, at 12 months, the difference in ALSFRS-R change between patients in the highest and lowest pre-diagnostic slope quartiles was 2.04 points in PARALS (95% CI 1.25–2.83) and 2.47 points in ERRALS (95% CI 1.07–3.86). However, the linear and quadratic interactions together increased the marginal *R*^2^ by only 0.43 percentage points in PARALS and 1.68 percentage points in ERRALS.

**Conclusions:**

The pre-diagnostic ALSFRS-R slope identifies meaningful average differences in subsequent decline between patient groups but provides limited information for predicting individual trajectories.

**Supplementary Information:**

The online version contains supplementary material available at https://doi.org/10.1007/s00415-026-14177-2.

## Introduction

Amyotrophic lateral sclerosis (ALS) is a rapidly progressive neurodegenerative disease affecting motor neurons and leading to death after a median survival of 2–3 years from symptom onset [1]. While survival varies widely across patients [1, 2], the rate of disease progression also appears to change substantially, both across patients and within the same patient over time [3]. Characterizing this variability remains challenging because no single metric reliably captures individual disease progression throughout the disease course [4].

The revised ALS functional rating scale (ALSFRS-R) is the most widely used measure of disease progression in ALS [5]. Its total score, collected at each clinical visit, is commonly used to derive several metrics of disease progression. Among these, the monthly rate of ALSFRS-R decline from symptom onset to the visit of interest is one of the most frequently adopted. In clinical practice, this metric is usually calculated at the time of diagnosis and is referred to as the pre-diagnostic ALSFRS-R slope (also known as preslope) [6].

The pre-diagnostic ALSFRS-R slope has consistently been shown to provide prognostic information about both subsequent functional decline and survival beyond established prognostic factors [6–9]. Accordingly, it has also been incorporated into the design of selected randomized clinical trials, both as an eligibility criterion and as a stratification variable during randomization, with the aim of reducing baseline heterogeneity [10].

In routine clinical practice, clinicians may use the pre-diagnostic slope to estimate an individual patient’s expected rate of functional deterioration after diagnosis and to counsel patients accordingly. However, the extent to which this metric provides incremental information for predicting subsequent functional trajectories across patients remains unclear.

In this study, we used two large population-based ALS cohorts to evaluate the association between the pre-diagnostic ALSFRS-R slope and functional decline over the subsequent 18 months and to quantify its incremental contribution to predict individual trajectories beyond established prognostic factors.

## Methods

Patients from the Piemonte and Valle d’Aosta Register for ALS (PARALS), a prospective population-based registry covering approximately 4.3 million inhabitants in northwestern Italy, served as the primary cohort [11]. All patients diagnosed between January 2000 and December 2022 with at least one ALSFRS-R assessment at the time of diagnosis were included. Patients fulfilling the revised El Escorial diagnostic criteria [12] are routinely followed at one of two tertiary ALS centers and undergo scheduled multidisciplinary assessments according to international recommendations [13]. Because the ALSFRS-R is collected at every visit, repeated functional evaluations are available for most patients during their follow-up. For the purpose of this study, visits occurring after tracheostomy were excluded, consistent with the assumption that tracheostomy was considered equivalent to death (see below).

For each patient, the pre-diagnostic ALSFRS-R slope (or pre-diagnostic FRS slope) was calculated as (48—ALSFRS-R total score at diagnosis) divided by the onset-to-diagnosis interval (months). Several post-diagnostic FRS declines were calculated separately at 6, 12, and 18 months after diagnosis using the visit closest to each target time point, as the change in ALSFRS-R total score from diagnosis divided by the follow-up interval (months).

The relationship between pre- and post-diagnostic functional decline was investigated through multiple complementary approaches. First, Pearson’s and Spearman’s correlation coefficients were calculated for each post-diagnostic time point to assess the extent to which the rate of progression before diagnosis was associated with that observed at 6, 12, and 18 months.

Then, to assess whether and to what extent the pre-diagnostic slope provided incremental information on the prediction of subsequent functional decline beyond established prognostic factors, multivariable linear regression models were built using the post-diagnostic slopes (at 6, 12 and 18 months) as outcomes. Base models included sex, age at onset, and site of onset (spinal vs bulbar), after which the pre-diagnostic slope was added as an additional predictor. For each time point, nested models were compared using an F test, and the improvement in explained variance (Δ*R*^2^) was quantified. Sensitivity analyses additionally adjusted for baseline forced vital capacity (FVC, expressed as percentage of the predicted value based on sex and age) and the percentage of body mass index (BMI) lost at diagnosis (BMI premorbid—BMI at diagnosis/time from onset to diagnosis *100). In these complete-case analyses, to compare the prognostic contributions of FVC, BMI and pre-diagnostic FRS slope, each predictor was first added separately to the basic clinical model. A full model including all three predictors was then fitted, and the unique contribution of each predictor was quantified as the reduction in *R*^2^ obtained after removing that predictor from the full model.

To evaluate whether the pre-diagnostic FRS slope was associated with the subsequent rate of functional decline while using all available repeated ALSFRS-R assessments during the same 18-month follow-up period, a complementary longitudinal analysis was performed using linear mixed-effects models. Repeated ALSFRS-R total scores were modeled as the outcome, and time was expressed in months from the diagnosis. Fixed effects included time, standardized pre-diagnostic FRS slope (mean = 0, standard deviation = 1), sex, age at onset centred at the cohort mean, and site of onset. Patient-specific random intercepts and random slopes for time were included to account for between-patient differences in both baseline functional status and longitudinal progression. Interactions between time and each covariate were included to allow these factors to be associated with the rate of post-baseline decline, rather than only with the baseline ALSFRS-R level. In particular, to assess whether the pre-diagnostic FRS slope was associated with subsequent ALSFRS-R decline, models with and without the time-by-pre-diagnostic slope interaction were compared using likelihood-ratio tests. The final model was refitted using restricted maximum likelihood, and the additional variance explained by the interaction was quantified through the change in marginal *R*^2^. The mixed-effects analysis was repeated in the subgroup of patients with available baseline FVC and BMI loss.

To assess whether the association between the pre-diagnostic FRS slope and subsequent ALSFRS-R progression varied nonlinearly over time (that is, whether the subsequent rate of decline could accelerate or decelerate over time rather than remain constant), the mixed-effects analysis was extended by adding a fixed quadratic term for time and its interaction with the standardized pre-diagnostic FRS slope. These terms allowed the rate of decline to accelerate or decelerate over time, with the degree of curvature depending on the pre-diagnostic slope. To determine whether these additional terms improved model fit, nested models were fitted using maximum likelihood and compared using likelihood-ratio tests, the Akaike information criterion (AIC), and the Bayesian information criterion (BIC). The selected model was subsequently refitted using restricted maximum likelihood. The pre-diagnostic slope was retained as a continuous variable in all models. Using the coefficients estimated by the selected model, we calculated the difference in ALSFRS-R change from diagnosis associated with a one-standard-deviation higher pre-diagnostic slope at 6, 12, and 18 months. To provide a more clinically interpretable comparison, we also used the model to estimate, at 6, 12, and 18 months, the difference in ALSFRS-R change between patients whose standardized pre-diagnostic slopes corresponded to the mean values observed in the highest and lowest quartiles. The combined contribution of the linear and quadratic interactions between the pre-diagnostic slope and time was quantified through the change in marginal *R*^2^ between the complete model and a model retaining the same quadratic time structure and the main effect of the pre-diagnostic slope, but excluding both interactions between the pre-diagnostic slope and time.

Finally, because death or tracheostomy could result in informative attrition from the longitudinal ALSFRS-R assessments, joint longitudinal-survival models were fitted as sensitivity analyses. The analysis included all eligible patients with a baseline ALSFRS-R assessment, and the longitudinal submodel included all ALSFRS-R total scores recorded from baseline through 18 months. The longitudinal submodel followed the specification of the selected quadratic mixed-effects model, except that patient-specific random intercepts and linear slopes were assumed to be independent. The survival submodel considered time from the baseline ALSFRS-R assessment to death or tracheostomy, with censoring at the earlier of 18 months or the last known follow-up. It included sex, centered age at onset, site of onset, and standardized pre-diagnostic FRS slope. The longitudinal and survival submodels were linked by allowing the risk of death or tracheostomy to depend on the patient’s estimated current underlying ALSFRS-R value. Joint models were estimated by maximum likelihood using a Weibull baseline hazard and pseudo-adaptive Gauss–Hermite quadrature. Estimates from the joint model were compared with those from the corresponding conventional mixed-effects model fitted in the same patients.

All analyses were independently replicated in the Emilia-Romagna Registry for ALS (ERRALS), a population-based registry covering approximately 4.5 million inhabitants in northeastern Italy [14]. Patients diagnosed between 2009 and 2023 were analyzed applying the same variable definitions and analytical pipeline.

Comparisons of medians (reported with IQRs) and proportions in demographical and clinical variables across phenotypes were performed using Mann–Whitney *U* test and chi-square test, respectively. Survival was calculated from the date of diagnosis to the earliest of tracheostomy, death, or censoring (June 30, 2026). A *p*-value < 0.05 was considered as statistically significant. All analyses were performed using *R* (version 4.5.112) [15].

This study was approved by the Ethical Committee of the Turin ALS Centre (Comitato Etico Azienda Ospedaliero-Universitaria Città della Salute e della Scienza, Torino, #0038876) and by the Comitato Etico Provinciale of Modena, file number 124/08, on 2 September 2008 (ERRALS data), and has been performed in accordance with the ethical standards laid down in the 1964 Declaration of Helsinki and its later amendments. The databases were anonymized according to Italian law for the protection of privacy. Patients provided written informed consent.

## Results

During the study period, a total of 2,895 patients were diagnosed with ALS in the PARALS registry. Of these, 2,222 (76.7%) underwent an ALSFRS-R evaluation at diagnosis and were included in the study (Table 1). Patients included in the study did not differ significantly from excluded patients with respect to the main demographic and clinical characteristics (supplementary Table 1). Median survival was slightly longer among excluded patients, possibly reflecting patients with slower disease progression who did not undergo an ALSFRS-R assessment at diagnosis but were subsequently evaluated during follow-up (21.1 months, 95% CI 18.8–23.2, in excluded patients versus 19.3 months, 95% CI 18.3–20.4, in included patients) although the difference was not statistically significant (log-rank *p*-value = 0.1). Patients included in the study were followed for a median of 5 visits (IQR 2–9) over a median follow-up of 371 days (IQR 98.8–751.3) from diagnosis. The median pre-diagnostic FRS slope was 0.62 points/month (IQR 0.32–1.18).

**Table 1:** Demographic and clinical characteristics of patients included in the study

	ERRALS (*n* = 1543)	PARALS (*n *= 2222)	*p*-value
Sex, M (%)	856 (55.5)	1222 (55.0)	0.796
Onset age, years (median [IQR])	67.2 (58.7–74.2)	67.8 (59.9–74.4)	0.216
Onset site, spinal (%)	1066 (69.1)	1442 (64.9)	0.008
Diagnostic delay, months (median [IQR])	10.0 (6.0–16.0)	9.0 (5.7–13.7)	0.014

*ERRALS* Emilia Romagna Registry for ALS, *PARALS* Piemonte and Valle d’Aosta ALS Registry, *IQR* interquartile rage

Of these patients, 957 (43.1%) had an ALSFRS-R evaluation at 6 months and showed a median FRS slope from diagnosis of 0.96 points/month (IQR 0.43–1.93), 685 (30.8%) had an ALSFRS-R evaluation at 12 months (median FRS slope of 0.92, IQR 0.43–1.41) and 472 patients (21.2%) had an ALSFRS-R evaluation at 18 months from diagnosis (median FRS slope from diagnosis 0.78, IQR 0.38–1.15). As expected, survival significantly differed across patients included in these four groups, configuring an informative attrition rate. Patients with an ALSFRS-R assessment at 6 months had a median survival of 22.4 months (95%CI 21.3–23.7) from diagnosis, compared with 15.8 months (95%CI 14.6–17.4) among those without a 6-month evaluation. Similar differences were observed at 12 months (26.7, 95%CI 25.2–28.8, vs 14.0, 95%CI 13.3–15.0 months) and 18 months (35.4, 95%CI 31.8–38.6, vs 15.0, 95%CI 14.3–15.7 months), with all comparisons being highly significant (log-rank *p* < 0.0001).

**Fig. 1:**
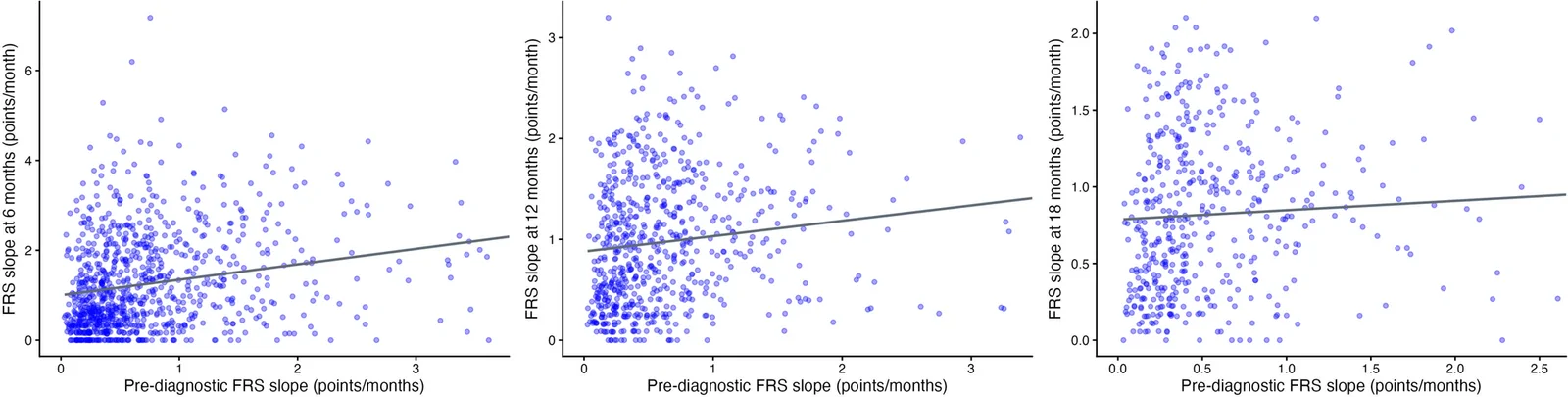
Scatter plots of pre-diagnostic versus post-diagnostic FRS slopes in the PARALS cohort

The pre-diagnostic ALSFRS-R slope showed a modest monotonic association with subsequent post-diagnostic decline (Spearman’s *ρ* = 0.19–0.30). The proportion of variance explained by the linear association remained low overall, being greatest at 6 months and progressively decreasing thereafter (*R*^2^ = 6.1–0.4%) (Table 2). This weak association was also evident from visual inspection of the scatter plots, which showed substantial dispersion of post-diagnostic slopes across the entire range of pre-diagnostic slopes at all time points (Fig. 1). Also, the limited association between pre- and post-diagnostic slopes did not appear to be primarily explained by the ALSFRS-R floor effect (that is, by the tendency of patients with lower baseline ALSFRS-R scores to have higher pre-diagnostic slopes but a more restricted range of observable post-diagnostic decline) as stratified analyses showed no consistent attenuation of the incremental contribution of the pre-diagnostic slope among patients with lower baseline ALSFRS-R scores (Supplementary Table 2).

**Table 2:** Pearson’s and Spearman’s correlation coefficients between pre-diagnostic and post-diagnostic FRS slopes in the PARALS cohort

Timepoint	*N*	Pearson’s *r* (95%CI)	*R*^*2*^	Pearson’s *p*-value	Spearman’s *ρ*	Spearman’s *p*-value
6 months	957	0.25 (0.19–0.30)	0.061	8.7*10^–15^	0.304	6.6*10^–22^
12 months	685	0.16 (0.08–0.23)	0.025	3.3*10^–5^	0.249	4.0*10^–11^
18 months	472	0.07 (− 0.02–0.15)	0.004	0.16	0.192	2.6*10^–75^

The regression models confirmed that adding pre-diagnostic FRS slope to a model already including sex, age at onset, and onset site only modestly increased the proportion of explained variance in the post-diagnosis FRS slope. At 6 months after diagnosis, although the improvement in model fit was statistically significant (nested-model F test, *p* = 3.9×10⁻^15^), the addition of pre-diagnostic FRS slope increased the explained variance by 6.1 percentage points (*R*^2^ from 2.7% to 8.8%). Such improvement decreased in the model predicting the FRS slope at 12 from diagnosis (Δ*R*^2^ = 2.8 percentage points, *R*^2^ from 2.8 to 5.6%, *p* = 8.2×10^⁻6^) becoming not statistically significant at 18 months from diagnosis (Δ*R*^2^ = 0.6 percentage points, *R*^2^ from 5.3 to 5.9%, *p* = 0.08). When baseline FVC and the percentage loss of BMI from premorbid to diagnostic evaluation were additionally included in the models in a subgroup of patients for whom these data were available (*n *= 888, 644 and 446 at 6, 12 and 18 months, respectively), the incremental contribution of the pre-diagnostic FRS slope became even smaller (5.2, 1.9, and 0.08 percentage points at 6, 12, and 18 months after diagnosis, respectively, being not statistically significant at 18 months, *p *= 0.5).

Notably, at 6 months the pre-diagnostic FRS slope accounted for more unique explained variance than FVC and BMI loss (5.2, 3.7, and 1.0 percentage points, respectively). However, at 12 months and 18 months, FVC provided the largest unique contribution (3.3 and 2.3 percentage points), while the loss of BMI contributed with 0.7 and 1.2 percentage points, respectively.

The complementary linear mixed-effects analysis included 1,743 patients and 9,670 ALSFRS-R assessments during the first 18 months after diagnosis (median of 5 assessments, IQR 3–7). A higher pre-diagnostic FRS slope was associated with faster subsequent decline: each one-standard-deviation increase corresponded to an additional monthly loss of 0.14 ALSFRS-R points (95% CI 0.10–0.19, likelihood-ratio test *p *= 9.8×10⁻^1^⁰). However, including this association increased the marginal *R*^2^ by only 0.6 percentage points, from 36.5% to 37.1%, indicating that the pre-diagnostic slope explained only a small additional proportion of the longitudinal variability beyond other prognostic factors considered. In the analysis including baseline FVC and premorbid-to-baseline BMI loss (1,538 patients and 8,892 ALSFRS-R evaluations), the time-by-pre-diagnostic slope interaction was attenuated but remained statistically significant (*β* = − 0.10, 95% CI − 0.15 to − 0.06; *p* = 8.2×10⁻⁶), increasing the marginal *R*^2^ by only 0.35 percentage points.

Adding a common quadratic time term to the linear mixed-effects model did not significantly improve model fit (likelihood-ratio *p *= 0.148). However, allowing the quadratic time effect to vary according to the pre-diagnostic FRS slope markedly improved model fit (*p *= 1.7×10⁻^12^), with reductions in both AIC (from 54,854 to 54,806) and BIC (from 54,962 to 54,921). Accordingly, a one-standard-deviation higher pre-diagnostic slope was associated with a faster initial decline (linear time-by-pre-diagnostic slope interaction: *β *= − 0.29 ALSFRS-R points/month, 95% CI − 0.35 to − 0.23) but also with subsequent relative deceleration (quadratic time-by-pre-diagnostic slope interaction: β=0.01 points/month^2^, 95% CI 0.01–0.02). Consequently, the estimated additional ALSFRS-R decline associated with a one-standard-deviation higher pre-diagnostic slope was 1.24 points at 6 months (95% CI 0.94–1.53), 1.43 points at 12 months (95% CI 0.88–1.99), and 0.59 points at 18 months (95% CI − 0.41 to 1.58). The corresponding differences between patients in the highest and lowest pre-diagnostic slope quartiles were 1.76 points (95% CI 1.34–2.18), 2.04 points (95% CI 1.25–2.83), and 0.84 points (95% CI −0.58 to 2.25), respectively. However, despite the improvement in model fit, the linear and quadratic interactions with the pre-diagnostic slope jointly increased the marginal *R*^2^ by only 0.43 percentage points, confirming its limited incremental contribution to explaining individual longitudinal variability.

In the joint longitudinal-survival analysis, adding a quadratic effect of time improved model fit compared with the linear specification (likelihood-ratio test: *p *= 0.009). Allowing the quadratic time effect to vary according to the standardized pre-diagnostic FRS slope produced a further substantial improvement (*p*<0.0001). The complete quadratic model was therefore strongly favored over the linear model (*p*<0.0001; AIC: 66,426.36 vs. 66,469.62; BIC: 66,551.86 vs. 66,583.71). In the complete model, the time-by-pre-diagnostic slope interaction was negative (*β *= −0.285, standard error = 0.027, *p *<0.0001), whereas the quadratic time-by-pre-diagnostic slope interaction was positive (*β *= 0.013, standard error = 0.0020, *p *< 0.0001). Accordingly, a one-standard-deviation higher pre-diagnostic slope was associated with an estimated additional ALSFRS-R decline of 1.25 points at 6 months and 1.58 points at 12 months, with the difference subsequently decreasing to 0.99 points at 18 months.

The ERRALS cohort included 1,582 patients, of whom 1,543 (97.5%) were available for analysis. (Table 1). The median pre-diagnostic FRS slope was 0.57 points/month (IQR 0.26–1.15). Of these, 551 (35.7%) had an ALSFRS-R evaluation at 6 months, with a corresponding median FRS slope of 0.57 points/month (IQR 0.16–1.38). A total of 413 (26.7%) and 293 (19.0%) patients had an ALSFRS-R evaluation at 12 and 18 months, respectively, with median FRS slopes of 0.69 points/month (IQR 0.26–1.24) and 0.62 points/month (IQR 0.26–1.06).

Spearman’s coefficients consistently indicated only modest monotonic associations (*ρ* = 0.23–0.24). The proportion of explained variance remained low throughout follow-up (*R*^2^ = 3.2%, 0.4%, and 0.4%, respectively), confirming the limited ability of the pre-diagnostic FRS slope to explain subsequent functional decline (supplementary Table 3).

In the linear mixed-effects specification, a one-standard-deviation higher pre-diagnostic FRS slope was associated with an additional monthly decline of 0.07 ALSFRS-R points (95% CI 0.01–0.13; likelihood-ratio *p *= 0.020). However, the full quadratic model provided a substantially better fit than the linear model (likelihood-ratio test *p*<0.0001), and the addition of the quadratic-time-by-pre-diagnostic-slope interaction further improved model fit compared with a model containing only the linear interaction (*p *< 0.0001; AIC: 39,131 vs. 39,249; BIC: 39,240 vs. 39,351).

In the quadratic model, the association between the pre-diagnostic slope and subsequent decline varied over time. A one-standard-deviation higher pre-diagnostic slope was not associated with a discernible difference in ALSFRS-R change at 6 months (estimated additional decline: − 0.15 points, 95% CI − 0.55 to 0.26), but was associated with an additional decline of 1.38 points at 12 months (95% CI 0.60–2.16) and 4.59 points at 18 months (95% CI 3.31–5.86). The corresponding differences between patients in the highest and lowest pre-diagnostic slope quartiles were − 0.26 points at 6 months (95% CI − 0.99 to 0.47), 2.47 points at 12 months (95% CI 1.07–3.86), and 8.18 points at 18 months (95% CI 5.90–10.46). As in PARALS, however, the linear and quadratic pre-diagnostic-slope-by-time interactions together increased the marginal *R*^2^ by only 1.68 percentage points, from 34.6 to 36.3%.

The quadratic joint longitudinal–survival model included 1,535 patients contributing 6,718 ALSFRS-R assessments from the ERRALS cohort. The joint model confirmed the non-linear association between the pre-diagnostic slope and subsequent progression, although its estimated magnitude was attenuated at longer follow-up. Among the same patients, the estimated additional decline associated with a one-standard-deviation higher pre-diagnostic slope decreased from 1.46 to 0.73 points at 12 months and from 4.95 to 3.41 points at 18 months after accounting for informative attrition.

## Discussion

Using two large population-based cohorts, we confirmed that the pre-diagnostic FRS slope is associated with subsequent post-diagnostic functional decline and accordingly provides prognostic information at the group level. In both cohorts, patients with higher pre-diagnostic slopes followed different subsequent trajectories, with estimated differences of 2.0 and 2.5 points in ALSFRS-R change at 12 months between patients in the highest and lowest pre-diagnostic slope quartiles in PARALS and ERRALS, respectively. In PARALS, accounting for informative attrition arising when death or tracheostomy precluded subsequent ALSFRS-R assessments had little effect on the estimates, whereas it attenuated the differences at longer follow-up in ERRALS, particularly at 12 and 18 months. Informative attrition may therefore partly explain the marked late separation between patients in the highest and lowest pre-diagnostic FRS slope quartiles observed in ERRALS but not in PARALS although it does not fully account for the difference between the two cohorts. The 18 month ERRALS estimate should nevertheless be interpreted cautiously because relatively few patients in the highest pre-diagnostic slope quartile remained under observation at longer follow-up (*n *= 54).

Although these results support the prognostic relevance of the pre-diagnostic slope at the group level [6, 7], our findings also suggest that it provides limited information to distinguish subsequent trajectories across individual patients. In the PARALS cohort, its incremental contribution beyond established prognostic factors peaked at approximately 6 percentage points of explained variance at 6 months and became negligible by 18 months. Moreover, the linear and quadratic pre-diagnostic-slope-by-time interactions together increased the marginal *R*^2^ by only 0.43 percentage points in PARALS and 1.68 percentage points in ERRALS. This indicates that despite a meaningful average difference between groups, the pre-diagnostic slope explained little of the heterogeneity in individual trajectories. In the model additionally adjusted for baseline FVC and BMI loss, its incremental contribution was further reduced to 0.35 percentage points.

Notably, in the PARALS analyses conducted separately at 6, 12, and 18 months, the pre-diagnostic FRS slope made its largest incremental contribution at 6 months, whereas baseline FVC showed a more sustained contribution at later time points. This pattern suggests that respiratory function may provide more persistent information about subsequent functional decline than a global rate of progression reconstructed from symptom onset. Nevertheless, even the models combining demographic characteristics, site of onset, the pre-diagnostic slope, FVC, and BMI loss explained only a limited proportion of the between-patient variability in subsequent functional decline.

These findings are consistent with previous literature, showing that ALS progression cannot be adequately summarized by a single linear trajectory [3, 9, 16–18], with a faster functional decline during the earlier phases of the disease followed by subsequent deceleration [9]. Our findings also confirm previous evidence that the pre-diagnostic FRS slope is associated with subsequent functional decline. This association was demonstrated using data from the PRO-ACT database, in which a moderate correlation was found between the pre- and post-baseline FRS slopes (*r *= 0.40), but was also observed in real-world data, as the one using data from the Japanese ALS Registry. In a cohort of 246 patients, the authors found an even weaker association between the rate of progression from symptom onset to a study-defined baseline, set at approximately 1.5 years after onset, and functional decline during the subsequent 12 months. The weighted agreement between progression categories at the two time points was 0.036, while the corresponding continuous correlations were also very low (*r *= −0.103; *ρ *= 0.006). Differences from our estimates may reflect differences in patient selection, the definition of baseline, and the timing of the functional assessments, while the smaller sample size may have reduced the precision of the estimates [19].

Another particularly relevant study was the recently published PRECISION-ALS study, which investigated the natural history of ALSFRS-R trajectories using data from multiple ALS cohorts, including the PARALS cohort. In particular, 1421 of the 2222 PARALS patients included in the present study (63.9%) were also included in that study. The findings of the two studies are therefore not fully independent. The study demonstrated that the delta-FRS calculated at the first ALSFRS-R assessment was associated with the subsequent functional trajectory and with survival [9]. However, whereas that study characterized the broader natural history of ALSFRS-R progression, our study extends this evidence by quantifying the limited contribution of the pre-diagnostic FRS slope to explain the heterogeneity of the future trajectory across individual patients.

Clearly, this study inherits several well-recognized limitations of the ALSFRS-R, particularly its multidimensional structure and the ordinal nature of its individual items, neither of which is fully captured when the scale is analyzed as a single total score [20], These limitations are intrinsic to the scale itself and therefore apply not only to the present study but also to most previous investigations using the ALSFRS-R.

Nevertheless, our study also has specific limitations that should be acknowledged. The main limitation is that only a proportion of patients with an available pre-diagnostic slope contributed to the analyses at 6 months, with this proportion progressively decreasing at longer follow-up intervals. This attrition was not random but was strongly associated with survival, thus possibly hampering our ability to evaluate the predictive performance of the pre-diagnostic slope among patients with the most aggressive disease. Although this limitation is inherent to longitudinal observational studies in ALS, we addressed it by fitting a joint longitudinal-survival model that explicitly accounted for the relationship between the underlying ALSFRS-R trajectory and the risk of death or tracheostomy.

The pre-diagnostic and post-diagnostic slopes both included the ALSFRS-R score at diagnosis and were therefore not statistically independent. However, because this score enters the two equations with opposite signs, any resulting mathematical coupling would be expected to attenuate, rather than generate, a positive association.

The pre-diagnostic FRS slope depends on the reported date of symptom onset, which is subject to recall bias. Consequently, the pre-diagnostic slope is itself measured with some degree of error, potentially attenuating the observed associations. However, the magnitude and direction of this error could not be estimated, and its potential impact on the observed associations remains uncertain.

Finally, although the findings were analysed in two independent cohorts, both were derived from Italian populations, which may limit their generalizability to other geographical and healthcare settings.

The present study also has important strengths. Most importantly, the central finding that the pre-diagnostic slope provides group-level prognostic information but limited contribution for individual prediction was replicated in a large independent external cohort. Furthermore, the population-based nature of both cohorts supports the applicability of our findings to routine clinical practice by reflecting the full spectrum of ALS severity, progression rates, and onset-to-diagnosis intervals encountered in unselected patients [21].

In conclusion, we confirmed that the pre-diagnostic FRS slope is associated with subsequent functional decline. However, considered alone, it provides limited incremental information with respect to the heterogeneity of post-diagnostic functional trajectory across patients, especially at later time points. These findings may inform both the design of clinical trials and the interpretation of the pre-diagnostic FRS slope in routine clinical practice.

## Supplementary Information

Below is the link to the electronic supplementary material.

Supplementary file1 (DOCX 121 KB)

## Data Availability

Anonymized data relating to this article will be made available by request from any qualified investigator, subject to approval from the Comitato Etico Azienda Ospedaliero-Universitaria Città della Salute e della Scienza, Torino and from the Comitato Etico Provinciale of Modena.
