# Supplementary material for: Pre-diagnostic ALSFRS-R slope provides limited incremental information about the variability in subsequent functional decline in amyotrophic lateral sclerosis: a population-based study
Source: J Neurol. 2026 Oct 8;273(10):651. doi: 10.1007/s00415-026-14177-2 (PMC13649887; doi:10.1007/s00415-026-14177-2)
Supplement: Supplementary file 1 [file 415_2026_14177_MOESM1_ESM.docx]

**Supplementary material**

**Supplementary table 1**. Baseline demographic and clinical characteristics of patients included in and excluded from the study in the PARALS cohort.

|  | **Overall PARALS cohort (n=2895)** | **Patients excluded (n=673)** | **Patients included (n=2222)** | **p-value** |
| --- | --- | --- | --- | --- |
| Sex, M (%) | 1590 (54.9) | 368 (54.7) | 1222 (55.0) | 0.921 |
| Onset age, years (median [IQR]) | 68.0 (60.0-74.4) | 68.6 (60.2-74.5) | 67.8 (59.9-74.4) | 0.229 |
| Onset site, spinal (%) | 1867 (64.5) | 425 (63.2) | 1442 (64.9) | 0.433 |
| Diagnostic delay, months (median [IQR]) | 9.1 (5.8-14.2) | 10.1 (6.1-15.2) | 9.0 (5.7-13.7) | 0.026 |

*PARALS* Piemonte and Valle d’Aosta ALS Registry, *IQR* interquartile range.

**Supplementary Table 2.** Pearson's correlation coefficients between pre-diagnostic and post-diagnostic FRS slopes in the PARALS cohort, stratified by tertiles of the ALSFRS-R total score at diagnosis.

|  | **6 months** | | | |  | | **12 months** | | | |  | | **18 months** | | | |  |
| --- | --- | --- | --- | --- | --- | --- | --- | --- | --- | --- | --- | --- | --- | --- | --- | --- | --- |
| **ALSFRS-R at diagnosis tertile** | **N** | **Pearson r** | **R2** | **p-value** | |  | **N** | **Pearson r** | **R2** | **p-value** | |  | **N** | **Pearson r** | **R2** | **p-value** | |
| Low | 319 | 0,276 | 0,076 | 5.71*10-7 | |  | 132 | 0,191 | 0,037 | 0.0278 | |  | 77 | 0,158 | 0,025 | 0.1690 | |
| Intermediate | 319 | 0,186 | 0,035 | 0.0008 | |  | 166 | 0,229 | 0,052 | 0.0030 | |  | 96 | 0,093 | 0,009 | 0.3691 | |
| High | 319 | 0,147 | 0,022 | 0.0085 | |  | 170 | 0,231 | 0,053 | 0.0024 | |  | 138 | 0,257 | 0,066 | 0.0023 | |

**Supplementary table 3.** Pearson's and Spearman's correlation coefficients between pre-diagnostic and post-diagnostic FRS slopes in the ERRALS cohort.

| **Timepoint** | **N** | **Pearson’s r (95%CI)** | **R^2^** | **Pearson’s**  **p-value** | **Spearman’s** ρ | **Spearman’s**  **p-value** |
| --- | --- | --- | --- | --- | --- | --- |
| 6 months | 532 | 0.18 (0.09-0.26) | 0.032 | 3.7*10^-5^ | 0.231 | 7.10*10^-8^ |
| 12 months | 405 | 0.06 (-0.04-0.16) | 0.004 | 0.231 | 0.223 | 5.64*10^-6^ |
| 18 months | 278 | 0.06 (-0.05-0.18) | 0.004 | 0.294 | 0.244 | 3.84*10^-5^ |

*ERRALS* Emilia-Romagna Registry for ALS.
